# Views and experiences of Malaysian older persons about falls and their prevention—A qualitative study

**DOI:** 10.1186/s12877-016-0274-6

**Published:** 2016-05-06

**Authors:** Annaletchumy Loganathan, Chirk Jenn Ng, Wah Yun Low

**Affiliations:** Department of Primary Care Medicine, Faculty of Medicine, University of Malaya, Kuala Lumpur, 50603 Malaysia; Department of Biomedical Science, Faculty of Science, Universiti Tunku Abdul Rahman, Kampar, 31900 Perak Malaysia; Dean’s Office, Faculty of Medicine, University of Malaya, Kuala Lumpur, 50603 Malaysia

**Keywords:** Aged, Older adults, Accidental falls, Qualitative study, Falls preventions, Falls interventions

## Abstract

**Background:**

Few studies on falls interventions have been conducted in South East Asia. Despite its population ageing rapidly, the acceptability of interventions among the older population in this region remains variable. This study aims to explore views and experiences regarding falls and their prevention among older persons at high risk of falls.

**Method:**

Sixteen individuals aged 60 years and over with at least one fall in the preceding 12 months were recruited from our Primary Care clinics. A qualitative study using semi-structured interviews among individuals and focus-groups was conducted. Thematic analyses were conducted on transcriptions of audio-taped interviews using the WeftQDA software. The interviews ceased when data saturation was achieved.

**Results:**

The three themes included older persons’ views on falls, help-seeking behaviour and views on falls interventions. Many older persons interviewed did not perceive falls as a serious problem, some reported a stigma surrounding falls, while others felt they had not sustained more serious injuries due to God’s grace. Older persons sought traditional medicine and other alternative treatments for pain relief and other fall-related symptoms. Accessibility of healthcare facilities often prevented older persons from receiving physiotherapy or eye tests.

**Conclusion:**

The delivery of complex interventions for a multifactorial condition such as falls in the older persons in our setting is inhibited by various cultural barriers, falls perceptions as well as logistic difficulties. Efforts to establish a multi-disciplinary intervention among our older population will need to include strategies to overcome these issues.

## Background

It is commonly cited that one in three community-dwelling individuals aged 65 years and above fall at least once over the course of a year [1, 2]. The incidence of falls among the older population appears lower in East Asian populations, with metanalyses on studies conducted among Chinese populations reporting rates of 14.7 to 34 % among older persons aged 65 years and above [3]. Few studies have been conducted on falls in older persons in the South East Asian region, which have its own unique culture and ethnic composition. A study performed among rural community dwellers in Malaysia found that 27 % of their older villagers fell at least once in the previous year [4].

Numerous studies have now demonstrated the benefits of a variety of primary and secondary fall prevention strategies including home-based or group exercise, home hazard modification, medication review, rehabilitation, fall education, and multi-factorial fall risk assessment have now been published [5]. However, many older persons with increased falls risk do not receive these interventions [6, 7]. Potential barriers to effective interventions include lack of knowledge about fall prevention interventions, such as being unaware of the type of exercises that can improve balance and prevent falls [8]. They may also perceive falls as not being a medical problem or serious enough to require a doctor [9], and they may lack awareness of the availability of fall prevention interventions [10]. In addition, they usually link falls to carelessness rather than home hazards [11]. In one study, 60 % of older Australian people rated their fall risk as low and considered fall prevention campaigns to be irrelevant to them [12]. Another issue in adopting fall prevention intervention, as observed among UK elderly with high risk of falls, is the perception that fall prevention advice is not practicable, but merely common sense to be used like any other information or advice [6]. Among older persons behaviours that act as a barrier to initiating fall prevention intervention are resistance to precautions needed to avoid falls and engaging in activities with a high risk of falling [13], and hiding falls from their children concealment of falls and fear of institutionalization [10].

Other predictors of poor fall prevention uptake among older persons with high risk of falls include healthcare professional factors [14]. In two qualitative studies, older persons have reported, as barriers to managing fall risks, that healthcare professionals (HCPs) lack awareness on fall intervention services and do not offer continuity of care for patients who report falls, fail to make relevant referrals, and lack knowledge on how to help older persons gain access to fall prevention intervention [9, 15].

Previous systematic reviews highlighting barriers to the implementation of research evidence in the management of falls in older persons were mostly conducted among predominantly Caucasian populations [16, 17]. Prior to the effective implementation of falls interventions in the South East Asian setting, it is important to understand the perceptions of our older population on falls and falls interventions, as these are likely to be influenced by differences in culture, health systems and economic status. We, therefore, conducted a qualitative study to explore the views and experiences of our older population on falls and their perceptions of interventions which may reduce falls.

## Methods

Qualitative [18], individual in-depth interviews (IDI) and focus-group discussions (FGD) were used to identify and explore views and experiences of older persons concerning falls. During the in-depth interviews, the researchers encouraged participants to talk about issues pertinent to the research question using open-ended questions. The FGD was a group interaction which encouraged participants to explore and clarify individual and shared perspectives. For the FGDs, we selected and grouped the participants based on their working and non-working background and same language spoken to ensure homogeneity. The study was conducted among older persons with a history of at least one fall in the preceding 12 months recruited from the Primary Care Clinics at University of Malaya Medical Centre (UMMC), a teaching hospital in greater Kuala Lumpur, Malaysia. Malaysia is an upper-middle income country in South East Asia with its population comprising of the major ethnic groups of Malay, Chinese and Indians. Purposive sampling was employed in order to include participants from a variety of age groups, ethnicity and gender. Written informed consent and socio-demographic information was obtained from all participants. This study had been approved by the University Malaya Medical Centre (UMMC) Medical Ethics Committee (Reference: 926.2).

Focus group discussions were conducted in groups of six to 11 participants who interacted and considered their views in the context of others’ views [18]. It ensured that the data is robust and rich. Individual in-depth interviews were conducted between a researcher and older persons who were unable to join an FGD because of time constraints and scheduling conflicts. In this study, fall was defined as “unexpected event associated with coming to rest on the ground, floor, or lower level” [19].

The semi-structured interview guide for the study (Table 1) was developed from literature reviews and clinical expert’s opinions. The IDIs and FGDs were conducted using the same interview guide. The interviews were carried out between August 2012 and January 2013. The participants gave written consent prior to their participation in IDIs and FGDs. The IDIs lasted for 30–50 min, while the FGDs lasted for 60–80 min. The interviewer would consistently probe for additional information based on the interview guide to encourage participants to divulge their views to a greater degree. No further interviews were conducted once data saturation was achieved.

**Table 1:** Interview guide for FGD and IDI

PREAMBLE: If you have experienced at least once fall in the past 12 months, please share your views and experiences about it and how you sought help afterward
• Can you share with me your thoughts on your fall experiences?
• Can you tell me what actions you took after your fall? (Probe: hospital, clinics, etc.)
• Would you consider your fall a serious event in your life? If yes, how? If no, why?
• Would you allow a home visit to identify potential hazards that might lead to falls? (Probe: remove loose rugs and carpets, reorganise furniture)
• What do you think about exercise pertaining to fall prevention? (Probe: Tai Chi, aerobic, walking or dancing)
• What do you think about using an assistive device? (Probe: walking stick, walking frame).
• Have you experienced any problems getting a referral from a doctor for fall prevention interventions? (Probe: eye appointment, physiotherapy or hearing test)
• Do your family, doctors, and hospital take part in managing your falls? (Probe: do you think they effectively responded to your reported fall event? How?)

All the interviews were conducted by LWY and AL in both the Malay and English languages. The participants were PCM’s outpatients. However, the researcher did not interview close acquaintances or colleagues to avoid potential participant response bias. The interviews were transcribed verbatim, checked for accuracy and quality by listening to the audio recording and checked against the transcript, before being exported into the WeftQDA (Version 1.0.1) qualitative software for data analysis using a thematic approach. Transcripts in the Malay language were first analysed in their original language. Selected quotes were later translated to English. The translated quotes were verified with other researchers to ensure translational accuracy.

Each interview allowed the researchers to modify the interview guide. Data were analysed after several interviews before the researchers proceed to the subsequent interviews. This allowed the researchers to explore the new issues that emerge in the subsequent interviews. The interviews conducted informed each other as we analysed the subsequent interviews. The information analysed helped to improve the subsequent interviews as we can incorporate in emerging themes. Initially, the researchers independently read through two interview transcripts for familiarisation to determine a coding framework. A whole transcript was analysed by assigning codes to phrase, sentence or paragraph that described the meaning of the text segment. Texts that had a similar meaning were assigned under the same code, while texts with different meaning were given a new code. Each transcript was thoroughly analysed for new meanings that emerged until there were no new code arising from the transcript. Disagreements about coding and themes dealt with through discussion among the researches until consensus was reached. The researchers constantly discussed and challenged each other on the data analysis to ensure that the data is reflecting accurately the participants’ views and experiences. The coding framework was developed from mapping the themes and the categories emerged in the analysis were used to code the remaining transcripts. New codes that emerged were added to the coding framework upon discussion with the research team. Themes were broader groups of unifying concepts regarding the subject of inquiry [20]. Researchers’ views on the interpretation of the data were constantly debated on in order to identify and address any potential biases to improve the credibility of the findings. The findings from the in-depth interviews and focus group discussion of the older participants were triangulated with that from healthcare professionals regarding the management of older individuals with older participant’s views about falls and published elsewhere [21].

## Results

### Socio-demographics and fall profiles of the participants

A total of 16 older persons participated in the study. Besides individual interviews (*n* = 2), one focus group was with retired older persons (*n* = 6) and the other two with working older persons (*n* = 3; *n* = 5). Their socio-demographic information and fall profiles are detailed in Table 2. Participants were predominantly female in their mid-60s and consisting of one Indian, four Malays and 11 Chinese. About half were college graduates and still working. Most were living with their family.

**Table 2:** Socio-demographic details of participants

Characteristics	Number (*n* = 20)	Range
Age (years)		62–71
Gender		
Male	4	
Female	12	
Ethnicity		
Malay	4	
Chinese	11	
Indian	1	
Falls in the past 12 months		
Once	8	
Recurrent	8	
Currently living with		
Alone	1	
Family	15	
Employment		
Retired	8	
Working	8	
Education		
Primary (completed 6 years)	2	
Secondary (completed 12 years)	5	
Tertiary (college/university)	9	

### Emerging themes

Three themes emerged from the analysis. These themes included perceived views on falls, help-seeking behavior and interventions for preventing falls. Details of the main themes and subthemes details are shown in Table 3.

**Table 3:** Perceived views on falls and the falls interventions by older persons with a high risk of falls when preventing falls

Theme	Categories
1. Views on falls	1. Thankful for getting away with minor injuries
	2. Falls are difficult to predict
	3. Falls are not serious and therefore do not need to be disclosed
	4. Falls lead to serious complication
	5. Falls are part of old age
2. Help-seeking behavior	1. Seeking traditional medicine
	2. Delay in seeking medical attention
3. Views on falls interventions	1. Exercise can prevent falls
	2. Issues with the use of walking aids
	3. Home hazards assessment
	3. Response to referrals

### Views on falls

#### Thankful for getting away with minor injuries

Participants who only sustained minor injuries after their falls used the word ‘luck’ or the phrase ‘thank God’ to describe how they felt about their fall event. Some felt that it was God’s grace that protected them from more serious injuries.

*“The injury was just minor. Maybe it was just good luck, you see. Maybe God loves me, so He protects me from a bad fall.”* (aged 70; Malay female)

*“(I was) …so lucky! I only hurt my back, (there was) nothing serious” (aged 79; Chinese male)*

#### Falls are difficult to predict

While the participants were concerned about the unpredictability of fall occurrences, one participant expressed that he did not know how his latest fall had happened.

*“I was sitting on the chair. I was quite stable, as usual. But I don’t know how I toppled over and fell, you know. I can’t explain that”* (aged 81; Indian male)

Another participant felt that falls were not preventable as she fell despite being aware of the small steps she had been negotiating.

*“I was aware of the small steps, but it just happened in the blink of an eye. The next thing I knew, I found myself on the ground. There was nothing I could do to prevent it (falls)”* (aged 64; Malay female)

#### Falls are not considered serious and therefore do not need to be disclosed

The participants did not view their falls as serious issues which needed to be disclosed to their doctor or family members. One participant did not tell her husband about a fall to prevent him from worrying. Another participant discounted his falls as he felt it was a minor issue and did not need any medical attention. He explained that doctors had more pressing issues.

*“I will keep quiet. If I tell my husband, he will get worried, and it might make his blood pressure go up.”* (aged 67; Malay female)

*“I didn’t tell anyone. This is nothing…. not so important… to (have to) report to the doctor. He also has so many other medical problems to see to”* (aged 68; Chinese male)

#### Falls lead to serious complications

Participants recognized that falls could lead to serious complications. Many participants expressed their concerns about potential fractures related to falling.

“*Falls can cause hip fractures, you know. It (the hip fracture) can make me bed ridden for the rest of my life.”* (aged 79; Chinese male)

“*I have osteoporosis. If I fall, my bones fracture easily”* (aged 68; Chinese female)

#### Falls are part of old age

Participants also believed that their falls happened because of old age.

*“I fell while praying, you know. I fell when I tried to sit up after bending over in the Muslim prayer position. But I said (to myself) never mind, I am old.”* (aged 70; Malay female)

### Help-seeking behaviour

#### Seeking traditional medicine

Many participants self-medicated with traditional remedies or sought traditional treatment instead of seeing a doctor as they had previously found traditional medications to be effective. One participant took his own concoction of Chinese herbs for his fall-related injury.

*“That fall led to this cut (showing his wound) on my forehead and it was bleeding. I used my own medicine to stop the bleeding, which I made myself, you know”* (aged 74; Chinese male)

One woman described applying an ointment and using traditional Malay massage to relief the swelling in her legs. She expressed satisfaction with the treatment she received:

*“I didn’t seek medical attention because I managed it (my injuries) by just applying some ointment on the swollen area. I also went for massage for my leg or ankle swelling. It always worked better for me.”* (aged 70; Malay female)

#### Delay in seeking medical attention

For many participants, they did not feel a need to seek medical attention immediately after sustaining their injuries. They felt that their injuries were not serious enough to require medical attention or that their injuries would heal with home remedies. The older persons would only seek treatment from a doctor when an injury persisted or worsened.

*“(At) First I never wanted to see a doctor, so I bandaged my hand myself, but I went after one week because the pain still persisted”* (aged 68; Chinese female)

### Views on falls interventions

#### Exercise can prevent falls

The participants were aware of the benefits of exercise in preventing falls. They were involved in a range of exercises such as walking, aerobics, and line dancing as well as traditional exercises such as Qigong, Yoga, and Tai Chi.

*“Exercise is good. I feel my balance has improved, (I am) more alert and ….(have) avoided falls”* (aged 68; Chinese female)

“*My daily walk keeps my legs fit and strong*” (aged 81; Indian male)

“*I join the aerobics and line dancing group after work, exercise made me more flexible. I had a bad fall at the car park there (hospital) but since (I am) more flexible I survived that fall without any fracture.* ” (aged 68; Chinese female)

#### Issues with use of walking aids

Participants were reluctant to use walking aids as they found it difficult to use a walking aid while carrying out their usual daily tasks. For example, one participant found that using a walking aid reduced her freedom of movement when working in her garden.

*“I don’t like walking aids, you know. How do you do your work in the garden with a stick? No, it stops me from moving freely.”*(aged 70; Malay female)

Some participants were concerned about potential stigmas with using walking aids. Some felt too embarrassed to use them, while others associated their use with being weak or frail.

*“I am embarrassed to use that (a walking stick). Unless I am over 70, then maybe I will use one”* (aged 62; Chinese female)

*“I still don’t need one; it will give me more problems. Maybe when my legs become too weak to stand, then it will be useful.”* (aged 66; Chinese female)

#### Home hazards assessments

The study participants did not agree with suggestions to modify their homes. They resented any interference with their furniture arrangements or the design of their houses. Some could not relate falls to environmental hazards, but felt they were just due to carelessness.

*“I think home modifications are not necessary. I don’t like interference from others. It is my house, I know best about things in my own home.”*(aged 74; Malay female)

*“I think it won’t help much..our homes are usually quite full of things. Basically, you don’t fall because of the furniture but you fall because of your carelessness”* (aged 71; Chinese female)

#### Response to referrals

The reactions of participants to referrals for fall interventions varied. One participant expressed frustration on the long waiting times for appointments.

*“It is quite difficult to get an eye or ear appointment here (the hospital). They may give you an appointment for next year”* (aged 63; Chinese female)

One man rejected his referral for physiotherapy due to issues with accessibility. He expressed that travelling to hospital at his age is difficult.

*“It would have been fine if I lived nearby, but I live quite far away, you see. It is not easy for an older man like me to travel to hospital for physiotherapy”* (aged 81; Indian male)

## Discussion

Our qualitative study was unique in its involvement of an ethnically diverse older population in South East Asia in which the acceptability of complex interventions had not previously been evaluated. We had found that our older population often credited God’s grace or luck for not sustaining more serious injuries. Many perceived falls as trivial or an inevitability of old age and therefore did not disclose their falls or seek medical attention. Others, however, were concerned about the lack of predictability and potentially serious consequences of falls. Our participants often used traditional home remedies or alternative medicine for their injuries. While many accepted that physical exercise reduced the risk of falls and were already trying various forms of exercises, our participants did not feel home modifications were necessary. Long waiting times for specialist referrals and transportation difficulties led to some participants declining or not complying with recommended interventions.

Our participants felt that luck was on their side or credited God’s grace if they avoided serious injuries after their falls. Previous studies involving South Asians have been reported to believe that falls occur because of God’s will [8]. A separate study involving older Chinese people living in England, suggest that their older Chinese participants view falls as a destiny or punishment [10]. Despite the availability of fall prevention interventions, belief in fate or a higher being in control may prevent our older persons from accepting interventions. Previous studies have suggested the education is effective in improving the uptake on interventions among the general population [22–24]. However, studies on the effect of education have not been conducted in our culturally diverse older population with large variations in education attainments.

Views on falls among the older persons we had interviewed were consistent with that reported by previously published studies, including falls as part of aging [6, 8, 25], falls being inevitable [8] and reluctance to report falls [12, 26]. This demonstrates that the perception of falls as an inevitable part of aging is a universal concept which crosses cultural borders. While underreporting of falls is also a common feature, the underlying motives may vary across cultures. The participants had felt that falls were a trivial matter and were not worth troubling others with. This is in contrast with the findings of other studies which had suggested that older persons intentionally concealed falls due to concerns regarding potential repercussions of being placed in institutional care [9]. Few older Malaysians currently live in institutional care as there is a societal expectation for adult children to care for their parents and other childless older relations within their home environment [27, 28]. This may change rapidly in the next two decades in view of the demographic transition that we are experiencing in terms of with a rapidly ageing population and a rapid reduction in family sizes.

In our study, the older persons interviewed used traditional home remedies and saw traditional healers to address their injuries, with then no consideration for seeking medical attention for subsequent fall prevention. They only sought conventional treatment from medical doctors if the pain from their injuries persisted. In a qualitative study, older Chinese persons with high risk of falls living in England also described a preference for Chinese and herbal medicines and seeking medical advice in their native country rather than attending an English hospital [9]. The rationale underlying this health-seeking behaviour in terms of avoidance of conventional treatment remains unclear. One possible reason may be that older persons were more comfortable with the traditional remedies which they were far more familiar with, and had been passed down through generations within their families. Their view of modern medicine may also be that shrouded by a fear of the unknown, or they may avoid modern medicine simply due to concerns about the financial implications or poor accessibility. Healthcare expenditure among our older population remains mainly out-of-pocket. While access to healthcare is now free for older persons at Ministry of Health facilities, this is vastly oversubscribed and many would choose to avoid these facilities due to a perception of that the care provided is of poor quality.

Many older persons in our study accepted that physical exercise reduced the risk of falls and were already trying various forms of exercises such walking, *Qigong*, *Tai Chi*, line dancing and aerobics. They realised that attaining old age is associated with increased vulnerability to muscle weakness and brittle bones, and would undertake exercise to combat the deterioration. Further, our study also revealed that involvement in either group or home exercise is preferred. In contrast, in one survey almost half of the older respondents from Manchester UK reported their unwillingness to consider group based strength and balance exercise [7]. Weather, alongside lack of interest, poor health, depression, weakness, fear of falling, shortness of breath, and low outcomes expectation had been cited as barriers to acceptance of exercise interventions [29]. Exercise intervention as part of a falls prevention programme should perhaps take into account the preference for group or home-based exercises rather than those based in a health centre, for instance. Furthermore, weather conditions in Malaysia differ with its equatorial climate being rather permissive of year-round outdoor group activities. The benefits and implementation of interventions using group-based, culturally appropriate exercises such as Tai Chi should therefore be evaluated specifically in our setting in future studies.

One issue highlighted by our study was that older persons perceived long waiting times for appointments and difficulties with transportation led to some participants’ reluctance to accept recommended interventions. In a previous qualitative study, primary care providers identified that the access difficulty from home to medical care limited the older person’s ability to attend physical therapy referrals [30]. This could be due to the limited availability of shuttle services in the hospital for older persons. The public transportation system in Malaysia has not kept pace with its rapid development, with point-to-point transportation being underdeveloped. Subsidized or affordable door-to-door transportation for the older person with reduced mobility remains limited, and is not easily accessible. Hospital shuttle services for those who are unable to navigate long hospital corridors in our now supersized healthcare facilities of 1000–2000 bed capacity currently do not exist. Structured falls referrals schemes may also help overcome the long waiting times currently associated with hospital-based treatment.

### Limitations

Participants in this study were recruited from a single hospital; hence the healthcare system barriers as a potential factor in the poor uptake of fall prevention interventions cannot be further explored. The interviews were conducted in the hospital where the participants were recruited; hence, the environment may have influenced them to give socially desirable responses despite being informed that their responses would not affect their medical care and being assured confidentiality. The researcher did not interview close acquaintances or colleagues to avoid potential participant response bias. Furthermore, the sample size is small (*n* = 16) and to reflex differences in the perceptions between man and woman and as well as the three ethnic group. The Socio-economic data of the participants was not included in the study to reflect on participants’ participation in the fall prevention activities. There was no member checking was conducted in this study to obtain participants’ feedback on the study findings. Nevertheless, this study has revealed important insights into factors that may influence the implementation of complex interventions in our cultural setting in terms of its many similarities with existing studies as well as culturally unique perspectives on the use of traditional remedies and the difficulties in accessing modern medical treatment. This information will be invaluable in informing the implementation of effective falls prevention strategies specifically and other complex multifaceted interventions in the management of chronic disease among our older population in general.

## Conclusions

The study identified various views on falls, help-seeking behaviour as well as logistic difficulties to effective delivery of falls interventions from the perspectives of older persons with increased risk of falls. The ethnic and cultural differences among older persons must be taken into account to tackle issues pertaining to falls prevention. Further studies should evaluate the methods of delivery of complex interventions for falls in the older persons in our setting through structured evidence-based falls interventions which should take into account accessibility issues.

### Availability of data and materials

The dataset(s) supporting the conclusions of this article are included within the article.

### Ethical approval

This study received ethical approval from the University Malaya Medical Centre (UMMC) Medical Ethics Committee (Reference: 926.2).
